# Contributions to the bryological knowledge of ASPA 125, Fildes Peninsula, King George Island

**DOI:** 10.1186/s40659-018-0178-3

**Published:** 2018-08-30

**Authors:** Diego Knop Henriques, Bárbara Guedes Costa Silva, Gustavo Emílio Zuñiga, Paulo Eduardo Aguiar Saraiva Câmara

**Affiliations:** 1 Departamento de Botânica, Universidade de Brasília, Campos Universitário Darcy Ribeiro, Asa Norte, Brasília, Distrito Federal 70910 Brazil; 2 Laboratorio de Fisiología y Biotecnología Vegetal, Universidad de Santiago de Chile, Facultad de Química y Biología, Casilla 40, Correo 33, Santiago, Chile

**Keywords:** Mosses, Bryophyta, Protected areas, Antarctica, Fossil Hill

## Abstract

**Background:**

With 29 Km^2^, the Fildes Peninsula is the largest ice free area in King George Island and probably in Antarctica. The region is house of six permanent bases including the only airport in the South Shetlands, which led to impacts on its original landscape and vegetation. In recognition for the need to protect natural values, an Antarctic Specially Protected Area (ASPA 125) was established in the region. Focused mostly on protecting the fossils, the ASPA also plays a role in protecting the vegetation but so far, the management plan for the area does not contain a list of moss species present there.

**Results:**

We provided an updated study and checklist of mosses present in ASPA 125. A key to species identification and photographs of main morphological features are also available in this paper. Also, six new occurrences are reported for Fildes Peninsula.

**Conclusion:**

Considering the scarce knowledge about specific local floras in Antarctica associated with highly impacted area, of which only a fraction is protected, it is suggested the necessity to invest in detailed sampling studies, as well as in a better understanding of the local floras interactions in Antarctica.

## Background

The Fildes Peninsula (62°08′ to 62°14′S and 59°02′ to 58°51′W), located southwest on King George Island is the largest ice free area in the South Shetland archipelago. Its vegetation is composed by mosses, lichens and only two species of flowering plants [*Deschampsia antarctica* E. Desv. and *Colobanthus quitensis* (Kunth) Bartl.]. The first and only comprehensive checklist of mosses for the region listed 40 species [5], including Ardley Island, but not much is known about species distribution and conservation status.

Fildes Peninsula is also probably one of the areas with higher human impact in the South Shetlands, as it houses six permanent Antarctic Bases (Chilean stations Escudero, Frei and Fildes, Chinese station Great Wall, Russian station Bellingshausen and Uruguayan station Artigas), plus many refuges and modules. Dirt roads also exist connecting them, where you can also find an airport and a small village (Villa de las Estrellas), which includes small facilities like houses, postal office and a school.

Due to its proximity with South America, the area has become a major hub for tourism, logistics, scientists and whoever is coming back and forth from Antarctica. This leads to a high permanent concern on the negative impacts of such activities and how to comply with the environmental protection protocols in the region.

During the IV Antarctic Treaty Consultative Meeting (ATCM) in 1966, an area of 1.8 Km^2^ in Fildes Peninsula was chosen to be protected. It was originally designated as SPA 12, and is now the Antarctica Specially Protected Area 125 (ASPA 125). Fildes Peninsula also houses another nearby ASPA in Ardley island (ASPA 150), created in 1991 [6].

According to Annex V of the Environment Protocol, an ASPA is created to “protect outstanding environmental, scientific, historic, aesthetic or wilderness values, any combination of those values, or ongoing or planned scientific research” (ATS 2016). The ASPA 125 was created due to its unique fossil composition, being probably the area with higher paleontological importance in Antarctica (ASPA 125 management plan, 2009). The region is divided on eight different regions or zones labeled as ASPA 125 a–h.

As the main goal of the area is to protect its fossil richness, not much has been done in order to better understand its floristic composition. As a result, the area management plan does not provide a list of the plant species present in the area, but only mentions the presence of 40 species of mosses, 175 lichens and 2 flowering plants. However, this list actually refers to a study area at the Fildes Peninsula and Ardley [5] and not to the ASPA at all, as a consequence the number and species composition of ASPA 125 remains unknown.

In this study we provide a list of all moss species occurring in Fossil Hill (ASPA 125a), providing a key to its identification and general comments for some taxa of the moss flora of the Fildes Peninsula.

## Methods

### Study area

Fossil Hill (ASPA 125a) is located at the south center of Fildes Peninsula, it is easily accessible by both Chinese Station and Chilean bases. It has an area of about 0.568 Km^2^ (ASPA 125 management plan, 2009), and its higher elevations are ca. 130 m a.s.l. [5]. The area includes two main elevations: Büdel Hill and Fossil Hill.

ASPA 125a is the second largest zone after ASPA 125c, which is the buffer zone surrounding the Bellingshausen glacier dome, but it presents a more heterogeneous habitat and has marked altitudinal gradient.

### Collections

Material was collected during the austral summer of 2017. Collections were made with the use of knife or by hand following the recommendations of Schofield [8]. Species were identified with the use of proper literature, especially Ochyra et al. [4]. Vouchers are preserved at herbarium UB [9] and classification system follows Goffinet et al. [3].

### Fossil Hill checklist and identification key

Since there is no record of mosses specifically for the area, the list (Table 1) and key presented here are based solely on our own collections.

**Table 1:** Checklist of moss species in ASPA 125a, Fossil Hill, Fildes Peninsula

**Fig. 1:**
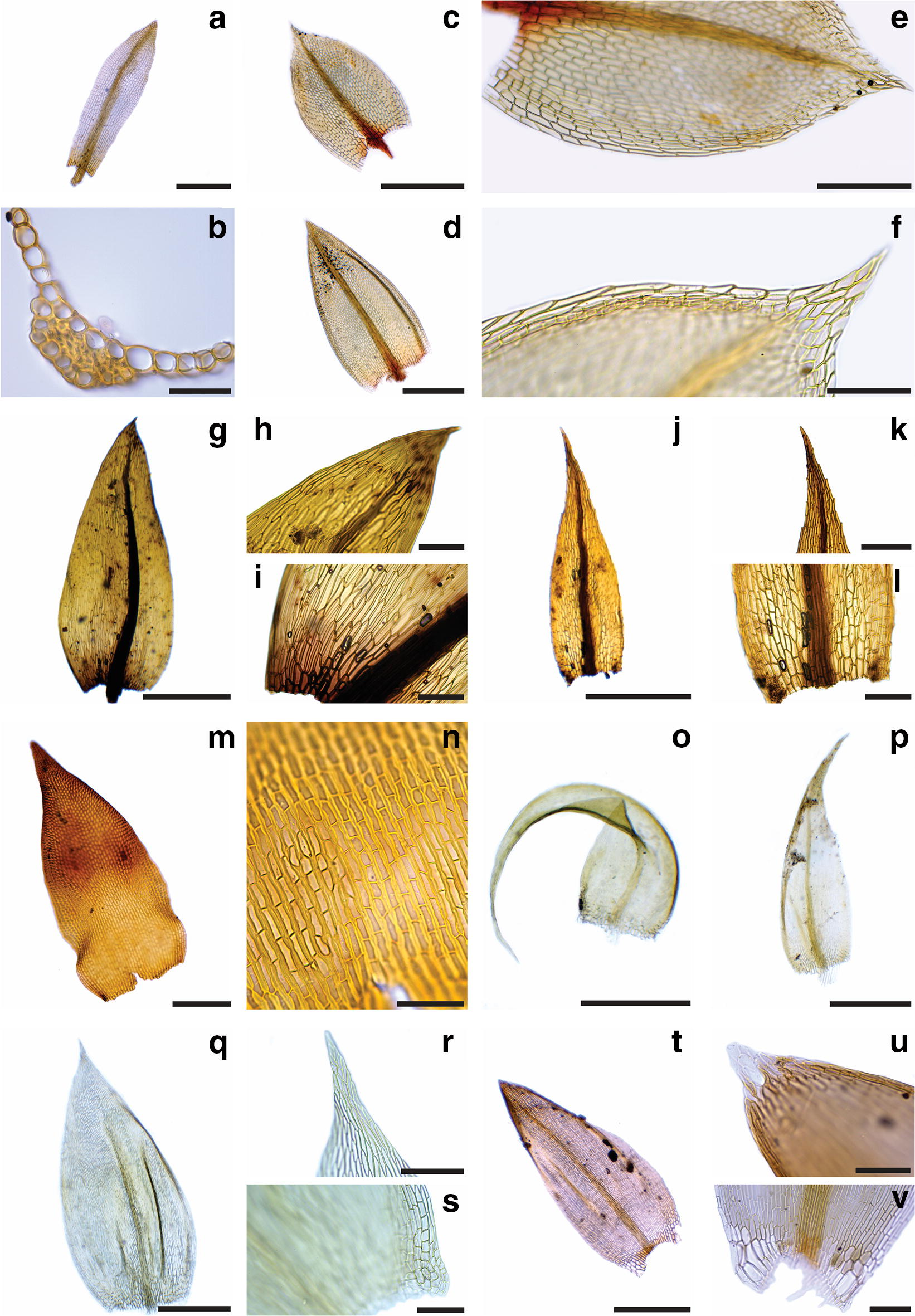
Morphology of some species of mosses found in ASPA 125 (Fildes Peninsula, King George Island). **a**, **b** *Meesia uliginosa*. **a** Leaf; **b** cross section of leaf. **c**–**f** *Bryum pseudotriquetrum.* **c**, **d** Leaves; **e** detail of leaf cells and margin; **f** detail of leaf apex. **g**–**i** *Pohlia cruda*. **g** Leaf; **h** detail of leaf apex and costa; **i** detail of leaf base. **j**–**l** *Pohlia nutans.* **j** Leaf; **k** detail of leaf apex and costa; **l** detail of leaf base. **m**–**n** *Andreaea gainii*. **m** Leaf; **n** detail of leaf cells, showing the thick walls. **o**–**p** *Sanionia uncinata.* Distinct leaves’ morphologies. **q**–**s** *Brachythecium austrosalebrosum.* **q** Leaf; **r** detail of leaf apex; **s** detail of the alar region. **t**–**v** *Warnstorfia sarmentosa.* **t** Leaf; **u** detail of leaf apex showing the colorless cells; **v** detail of leaf base and alar region. Scales: **a**, **c**, **d**, **g**, **j**, **o**, **p**, **q** and **t** 500 μm; **e**, **k** and **m** 200 μm; **f**, **h**, **i**, **l**, **r**, **s** and **v** 100 μm; **n** and **u** 50 μm

**Fig. 2:**
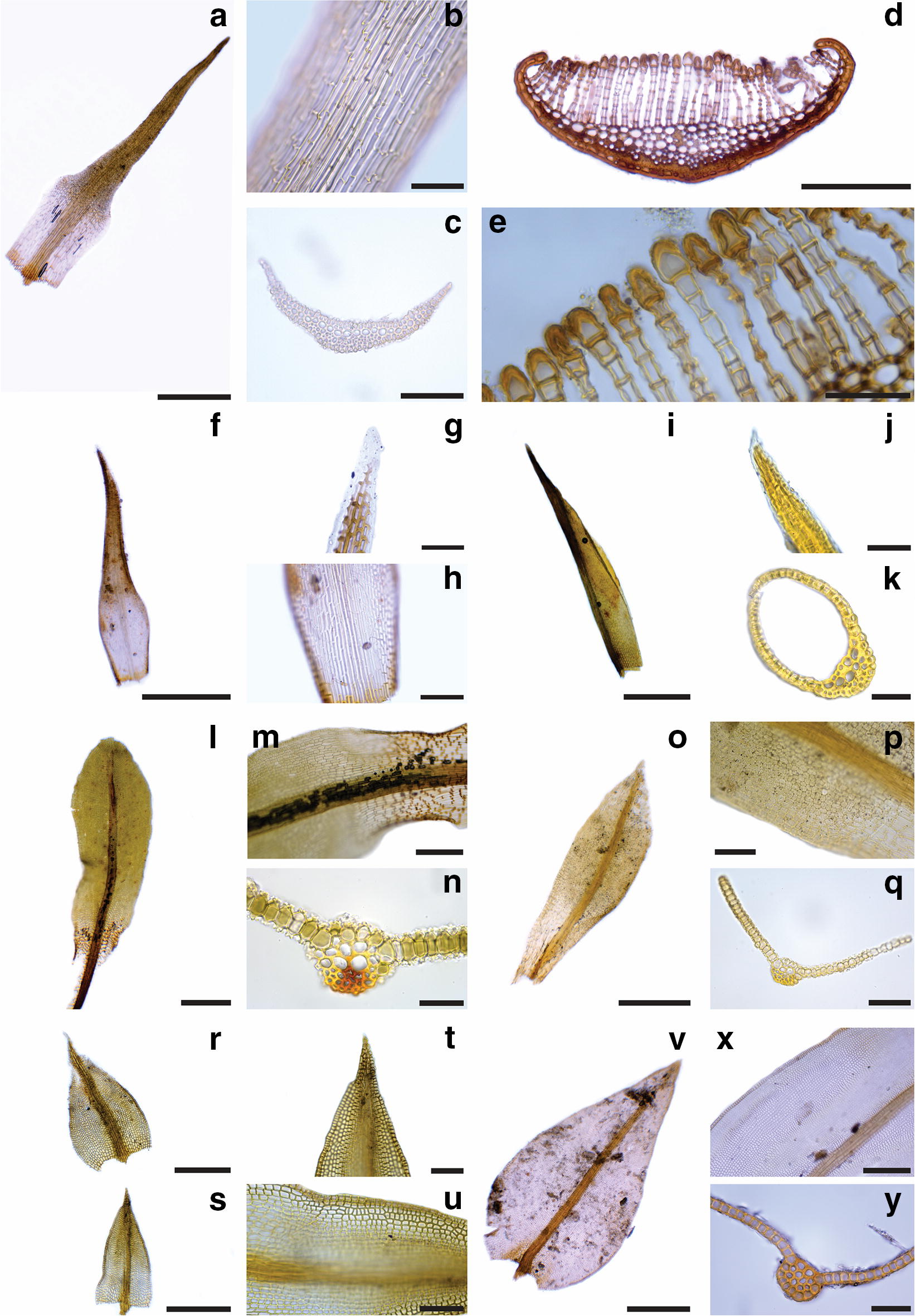
Morphology of some species of mosses found in ASPA 125 (Fildes Peninsula, King George Island). **a**–**c** *Bartramia patens*. **a** Leaf; **b** detail of papillae in the subula; **c** cross section of the leaf showing costa anatomy. **d**–**e** *Polytrichastrum alpinum.* **d** Cross section of leaf showing the lamellae; **e** detail of lamellae apical cells with thick walls. **f**–**h** *Ditrichum hyalinum*. **f** Leaf; **g** detail of leaf apex; **h** detail of leaf base cells. **i**–**k** *Distichium inclinatum.* **i** Leaf; **j** detail of leaf apex; **k** Cross section of leaf showing costa anatomy. **l**–**n** *Encalypta procera*. **l** Leaf; **m** detail of leaf cells in the transition from base to mid region; **n** cross section of leaf showing costa anatomy. **o**–**q** *Hennediella heimii.* **o** Leaf; **p** detail of leaf cells in the transition from base to mid region; **q** Cross section of leaf showing costa anatomy. **r**–**u** *Ceratodon purpureus.* **r**–**s**. Distinct leaves; **t** detail of leaf apex; **u** detail of margin and median leaf cells. **v**–**y** *Schistidium rivulare.* **v** Leaf; **x** detail of leaf cells in the mid region; **y** cross section of leaf showing costa anatomy. Scales **a**, **f**, **i**, **l**, **o**, **r**, **s** and **v** 500 μm; **d**, **m** and **x** 200 μm; **c**, **h**, **p**, **q**, **t** and **u** 100 μm; **b**, **e**, **g**, **j**, **k**, **n** and **y** 50 μm

Moss families and their respective species
Amblystegiaceae
*Sanionia uncinata* (Hedw.) Loeske (Fig. 1o, p)
Andreaeaceae
*Andreaea gainii* Cardot (Fig. 1m, n)
Bartramiaceae
*Bartramia patens* Brid. (Fig. 2a–c)
Brachytheciaceae
*Brachythecium austrosalebrosum* (Müll. Hal.) Kindb. (Fig. 1q–s)
Bryaceae
*Bryum archangelicum* Bruch & Schimp.^a^
*Bryum argenteum* Hedw.^a^
*Bryum pseudotriquetrum* (Hedw.) G. Gaertn., B. Mey. & Scherb. (Fig. 1c–e)
Calliergonaceae
*Warnstorfia sarmentosa* (Wahlenb.) Hedenäs (Fig. 1t–v)
Ditrichaceae
*Ceratodon purpureus* (Hedw.) Brid. (Fig. 2r–u)
*Distichium inclinatum* (Hedw.) Bruch & Schimp.^a^ (Fig. 2i–k)
*Ditrichum hyalinum* (Mitt.) Kuntze (Fig. 2f–h)
Encalyptaceae
*Encalypta procera* Bruch (Fig. 2l–n)
Grimmiaceae
*Schistidium andinum* (Mitt.) Herzog^a^
*Schistidium rivulare* (Brid.) Podp. (Fig. 2v–y)
Meesiaceae
*Meesia uliginosa* Hedw. (Fig. 1a, b)
Mniaceae
*Pohlia cruda* (Hedw.) Lindb. (Fig. 1g–i)
*Pohlia nutans* (Hedw.) Lindb. (Fig. 1j–l)
Polytrichaceae
*Polytrichastrum alpinum* (Hedw.) G.L.Sm. (Fig. 2d, e)
Pottiaceae
*Hennediella heimii* (Hedw.) R.H.Zander^a^ (Fig. 2o–q)
*Syntrichia caninervis* Mitt.^a^
*Syntrichia saxicola* (Cardot) R.H.Zander

Taxa are systematically organized according to Goffinet et al. [3]. Species photographs are indicated in parenthesis

^a^Indicates new occurrence for Fildes Peninsula

### Illustration

Specimens were dissected and important morphological characteristics were photographed under light microscope using a coupled camera to capture the images. Those were edited and assembled in two plates to illustrate the species (Figs. 1 and 2).

### Fildes Peninsula checklist

We used the checklist provided by Peter et al. [5] and compiled data from Ochyra et al. [4] as base for the list. We also checked for taxonomic updates, novelties and errors, resulting in the exclusion of some names. After that, we added data from our collections and updated the list for Fildes Peninsula (Table 2), following the classification system of Goffinet et al. [3]. For the family Pylaisiaceae, we followed the current concept of Câmara et al. [1].

**Table 2:** Updated checklist for Fildes Peninsula following the classification system of Goffinet et al. [3]

Moss families and their respective species
Amblystegiaceae
*Campylium polygamum* (Schimp.) Lange & C.E.O.Jensen
*Cratoneuropsis relaxa* (Hook.f. & Wilson) M.Fleisch. ex Broth. subsp. minor (Wilson & Hook. f.) Ochyra
*Sanionia georgico*-*uncinata* (Müll.Hal.) Ochyra & Hedenäs
*Sanionia uncinata* (Hedw.) Loeske
Andreaeaceae
*Andreaea depressinervis* Cardot
*Andreaea gainii* Cardot
*Andreaea regularis* Müll. Hal.
Bartramiaceae
*Bartramia patens* Brid.^a^
*Conostomum magellanicum* Sull.^a^
Brachytheciaceae
*Brachythecium austrosalebrosum* (Müll. Hal.) Kindb.
*Sciuro*-*hypnum glaciale (*Schimp.) Ignatov & Huttunen
Bryaceae
*Bryum archangelicum* Bruch & Schimp.^b^
*Bryum argenteum* Hedw.^b^
*Bryum orbiculatifolium* Card. & Broth.
*Bryum pallescens* Schleich. ex Schwägr.
*Bryum pseudotriquetrum* (Hedw.) G. Gaertn., B. Mey. & Scherb.
Calliergonaceae
*Warnstorfia fontinaliopsis* (Müll. Hal.) Ochyra
*Warnstorfia sarmentosa* (Wahlenb.) Hedenäs
Dicranaceae
*Chorisodontium aciphyllum* (Hook.f. & Wilson) Broth.
*Kiaeria pumila* (Mitt.) Ochyra
Ditrichaceae
*Ceratodon purpureus* (Hedw.) Brid.
*Distichium capillaceum* (Hedw.) Bruch & Schimp.
*Distichium hyalinum* (Mitt.) Kuntze
*Ditrichum inclinatum (Hedw.)* Bruch & Schimp. ^b^
Encalyptaceae
*Encalypta procera* Bruch
Grimmiaceae
*Bucklandiella sudetica* (Funck) Bednarek-Ochyra & Ochyra
*Schistidium andinum* (Mitt.) Herzog ^b^
*Schistidium antarctici* (Cardot) L.I.Savicz & Smirnova
*Schistidium rivulare* (Brid.) Podp.
*Schistidium urnulaceum* (Müll. Hal. in Neum.) B. G. Bell. ^a^
Meesiaceae
*Meesia uliginosa* Hedw.
Mniaceae
*Pohlia cruda* (Hedw.) Lindb.
*Pohlia nutans* (Hedw.) Lindb.
Orthotrichaceae
*Muelleriella crassifolia* (Hook.f. & Wilson) Dusén
Polytrichaceae
*Polytrichastrum alpinum* (Hedw.) G.L.Sm.
*Polytrichum piliferum* Hedw.
*Polytrichum juniperinum* Hedw.
*Polytrichum strictum* Menzies ex Brid.
Pottiaceae
*Didymodon brachyphyllus* (SuIl.) R.H.Zander
*Hennediella heimii* (Hedw.) R.H.Zander ^b^
*Syntrichia caninervis* Mitt.^b^
*Syntrichia filaris* (Müll.Hal.) R.H.Zander
*Syntrichia magellanica* (Mont.) R.H.Zander
*Syntrichia saxicola* (Cardot) R.H.Zander Pylaisiaceae *Roaldia revoluta* (Mitt.) P.E.A.S.Câmara & M.Carvalho-Silva
Seligeriaceae
*Holodontium strictum* (Hook.f. & Wilson) Ochyra
*Hymenoloma antarcticum* (Müll. Hal.) Ochyra
*Hymenoloma crispulum* (Hedw.) Ochyra

^a^Marks species with comments

^b^marks new occurrence for the Peninsula

## Results

### Species on Fossil Hill

Twenty-one species divided in thirteen families of mosses are recorded for the ASPA 125a site (Table 1). This represents about 41% of what is reported for the Fildes Peninsula [5] and about 18% of the whole Antarctic moss flora. Considering the relatively small area of the site, it shows its high importance and the relevance of the site in protecting the vegetation.

### Key to the moss species present at Fossil Hill

1. Plants Pleurocarpous … 2

1. Plants Acrocarpous … 4

2. Costa extending into apex, leaves strongly falcate … *Sanionia uncinata*

2. Costa ending below apex, leaves non falcate … 3

3. Leaves plane, oblong-ovate with rounded apex … *Warnstorfia sarmentosa*

3. Leaves strongly concave, ovate-lanceolate with acute to long-acuminate apex … *Brachythecium austrosalebrosum*

4. Costa absent … *Andreaea gainii*

4. Costa present … 5

5. Leaves with ventral lamellae in cross section … *Polytrichastrum alpinum*

5. Leaves without ventral lamellae in cross section … 6

6. Cells at leaf base differentiated, hyaline … 7

6. Cells at leaf base not differentiated, non hyaline … 11

7. Leaves with subula … *Batramia patens*

7. Leaves without subula … 8

8. Basal cells thick walled … *Encalypta procera*

8. Basal cells thin walled … 9

9. Transition from hyaline basal cells to chlorophyllose upper cells gradual, leaf base without border … *Hennediella heimii*

9. Transition from hyaline basal cells to chlorophyllose upper cells abrupt, leaf base with narrow border … 10

10. Costa percurrent, rarely short-excurrent, absence of hyaline hair point, leaves lanceolate to oblong-lanceolate, apex narrowly acute … *Syntrichia saxicola*

10. Costa always excurrent, presence of hyaline hair point, leaves ovate to ovate-lingulate, apex obtuse … *Syntrichia caninervis*

11. Leaves tightly appressed or crisped when dry; capsules immersed … 12

11. Leaves usually erect to erect-spreading, rarely crisped when dry; capsules emerged … 13

12. Leaf margins entire, apex forming a hair point … *Schistidium andinum*

12. Leaf margins serrulate to denticulate in the apex, apex not forming a hair point … *Schistidium rivulare*

13. Leaves distichous or not; leaf cells subquadratic, small lumen, thick walled … 14

13. Leaves never distichous; leaf cells hexagonal or fusiform, lax, thin walled … 16

14. Leaves always distichous … *Distichium inclinatum*

14. Leaves never distichous … 15

15. Leaves often forming a sheathing base, abruptly narrowed forming a long subula, leaf margins plane at least in the base … *Ditrichum hyalinum*

15. Leaves not forming sheathing base nor subula, leaf margins often strongly recurved throughout whole lamina … *Ceratodon purpureus*

16. Leaf apex shortly acute to obtuse, laminal cells quadratic to rectangular throughout … *Meesia uliginosa*

16. Leaf apex acute to apiculate, laminal cells hexagonal or fusiform … 17

17. Presence of leaf border … 18

17. Absence of leaf border … 20

18. Plants whitish, leaf base yellowish or green, costa subpercurrent … *Bryum argenteum*

18. Plants never whitish, leaf base red, costa percurrent to excurrent … 19

19. Costa percurrent to short-excurrent, leaves decurrent, acute to short acuminate … *Bryum pseudotriquetrum*

19. Costa long-excurrent, leaves non-decurrent, commonly long-acuminate … *Bryum archangelicum*

20. Costa brownish to red near leaf base, cells thin walled, upper laminal cells vermicular … *Pohlia cruda*

20. Costa dark green to brownish near leaf base, cells thick walled, upper laminal cells elongate-hexagonal … *Pohlia nutans*

### Species for Fildes Peninsula

According to data presented here, together with previous reports for the same location, there are 48 species of mosses divided in 17 families in the Fildes Peninsula (Table 2). This represents about 43% of all mosses known to Antarctica [4].

### Comments

*Batramia patens* Brid. It was only reported in Fildes for lake Kitiesh, but it is much more widely distributed in the Peninsula, including areas like Chilean Collins refuge, Uruguayan base Artigas, Fossil Hill and Chinese station Great Wall and its surroundings, being actually quite frequent. Putzke and Pereira [7] also reported it for Fildes, but the exactly location was not mentioned. Unfortunately we did not have the chance to study this material.

*Conostomum magellanicum* Sull. This species is not mentioned by Ochyra et al. [4] as occurring in Fildes Peninsula. We also did not collect it during our expedition and its report was maintained since it is present in Peter et al. [5].

*Schistidium urnulaceum* (Müll. Hal.) B.G. Bell. This species is not mentioned by Ochyra et al. [4] as occurring in Fildes Peninsula. We also did not find any sample of this taxon on our collections. Like *C. magellanicum,* it was maintained based on Peter et al. [5].

### Final considerations

Mosses are the second largest group of land plants, second only by Angiosperms [3]. In Antarctica, where there is only two species of native Angiosperms, mosses are the dominant vegetation, being present in both continental and maritime Antarctica.

Mosses are well known as bioindicators and for producing chemicals with pharmacological potential [2]. Unfortunately, not much is known about its ecological role and conservation status, especially in Antarctica. Very few ASPA management plans have a comprehensive list of moss species in their area, limiting more complex studies regarding the group in these places.

Fossil Hill with only 0.568 Km^2^ contains almost 20% of all moss diversity in Antarctica. The Fildes Peninsula with about 1400 Km^2^ contains 43% of all moss diversity in Antarctica. The knowledge of these areas continues to expand, as six new occurrences were found in this study.

The low level of understanding of the local flora associated with a highly impacted area of which only a fraction is protected highlights the necessity to invest in broader and detailed sampling studies, as well as in a better understanding of the floras in specific areas, both protected and not.

Antarctica is a fragile system and the Fildes Peninsula is already a highly impacted place. It can be expected that changes in climate conditions associated with local human activities may disturb the flora, reinforcing the need of understanding the true moss diversity of this region and its relation to other sites in South Shetlands.

## Abbreviations

ATCM: Antarctic Treaty Consultative Meeting
SPA: Special Protected Area
ASPA: Antarctic Specially Protected Area
a.s.l: above sea level
Fig: figure
